# Dataset of directional room impulse responses for realistic speech data

**DOI:** 10.1016/j.dib.2024.110229

**Published:** 2024-02-22

**Authors:** Stefan Fragner, Lukas Pfeifenberger, Martin Hagmüller, Franz Pernkopf

**Affiliations:** Signal Processing and Speech Communication Laboratory, Graz University of Technology, Austria

**Keywords:** Reverberant speech data, Speech processing, Room impulse response, Deep learning, Artificial intelligence

## Abstract

Obtaining real-world multi-channel speech recordings is expensive and time-consuming. Therefore, multi-channel recordings are often artificially generated by convolving existing monaural speech recordings with simulated Room Impulse Responses (RIRs) from a so-called shoebox room [1] for stationary (not moving) speakers. Far-field speech processing for home automation or smart assistants have to cope with moving speakers in reverberant environments. With this dataset, we aim to support the generation of realistic speech data by providing multiple directional RIRs along a fine grid of locations in a real room. We provide directional RIR recordings for a classroom and a large corridor. These RIRs can be used to simulate moving speakers by generating random trajectories on that grid, and quantize the trajectories along the grid points. For each matching grid point, the monaural speech recording can be convolved with the RIR at this grid point. Then, the spatialized recording can be compiled using the overlap-add method for each grid point [2]. An example is provided with the data.

Specifications Table

**Table:** 

Subject	Signal Processing
Specific subject area	Room Impulse Response; Speech Processing; Machine Learning
Data format	Raw; Computed
Type of data	Indexed Room Impulse Response Data
Data collection	The RIRs were computed from recorded (exponential) frequency sweeps. The sweeps were played by a 5W broadband loudspeaker (Visaton FR-58) that was mounted on top of a wheeled robot. The robot autonomously moves to every point in a grid and plays the sweep towards each of the 4 cardinal directions by rotating the speaker. More details on the robot can be found in the section: Experimental design, materials and methods. The recordings were taken using a circular 16-channel microphone array. The microphone array is custom built, uses the microphones Primo EM172-Z1 and phantom power adapters Audix APS910. These are connected to the microphone preamplifier and AD-converter (Focusrite OctoPre Mkii Dynamic). The AD-converter is connected (via ADAT cable) to the sound card (RME RayDat, Linux PC) and synchronized using Wordclock. The chirp was transmitted from the PC to the speaker using the Sennheiser ew1000Gs. The transmitter on the robot is connected to an amplifier (SMSL SA-36A pro) and then to the speaker.
Data source location	Institution: Graz University of Technology City/Town/Region: Graz Country: Austria
Data accessibility	Repository name: TU Graz Repository Library & Archives Data identification number: 10.3217/f6q9h-fc803 Direct URL to data: https://doi.org/10.3217/f6q9h-fc803
Related research article	Fragner, S., Topar, T., Giller, M., Pfeifenberger, L., Pernkopf, F. (2021) Autonomous Robot for Measuring Room Impulse Responses. Proc. Interspeech 2021, 2341-2342 https://www.isca-speech.org/archive/pdfs/interspeech_2021/fragner21_interspeech.pdf The related research article was peer-reviewed.

## 1 Value of the Data

• The dataset is useful for developing algorithms for speech enhancement, speaker separation and other speech applications. The RIRs enable the generation of more realistic multi-channel recordings for far-field speech proccessing applications. This overcomes the limitations of using simulated environments.
• Researchers will benefit from this dataset to obtain more realistic (multi-channel) speech data. This facilitates the development of more robust algorithms in the field of artificial intelligence, in particular speech processing. Especially for generating speech data with moving and / or multiple speakers.
• The provided software tools can be used for generating speech samples of moving speakers along a trajectory.

## 2 Background

Real-world multi-channel recordings are required to develop more robust algorithms in the fields of speech enhancement, speaker separation and far field speech recognition. However obtaining such data is very expensive and time-consuming. A reasonable approach is to use a dataset of real-world RIRs for convolving the clean audio samples of available speech datasets to artificially create such data. The objective was to provide such a RIR dataset for research in speech processing.

## 3 Data Description

The dataset contains RIRs for two different rooms, a classroom and a corridor.

**Fig. 1:**
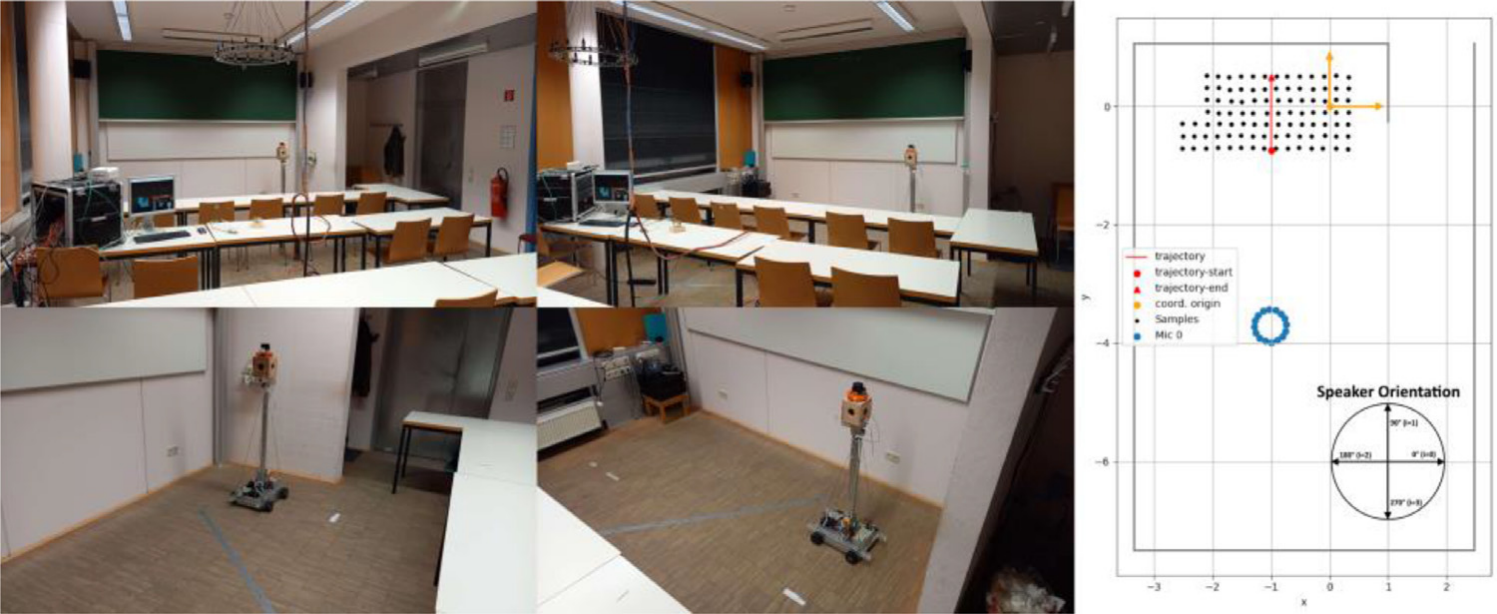
Classroom with autonomous robot (left), Room layout with RIR measurement locations and microphone positions (right).

In the classroom the grid of room locations has a spacing of 20 cm, with 97 different recording positions resulting in a total of 388 RIRs. The classroom is shown in Fig. 1 with the autonomous robot (left) and the geometric layout of the room (right) including the speaker orientation and the origin of the map.

**Fig. 2:**
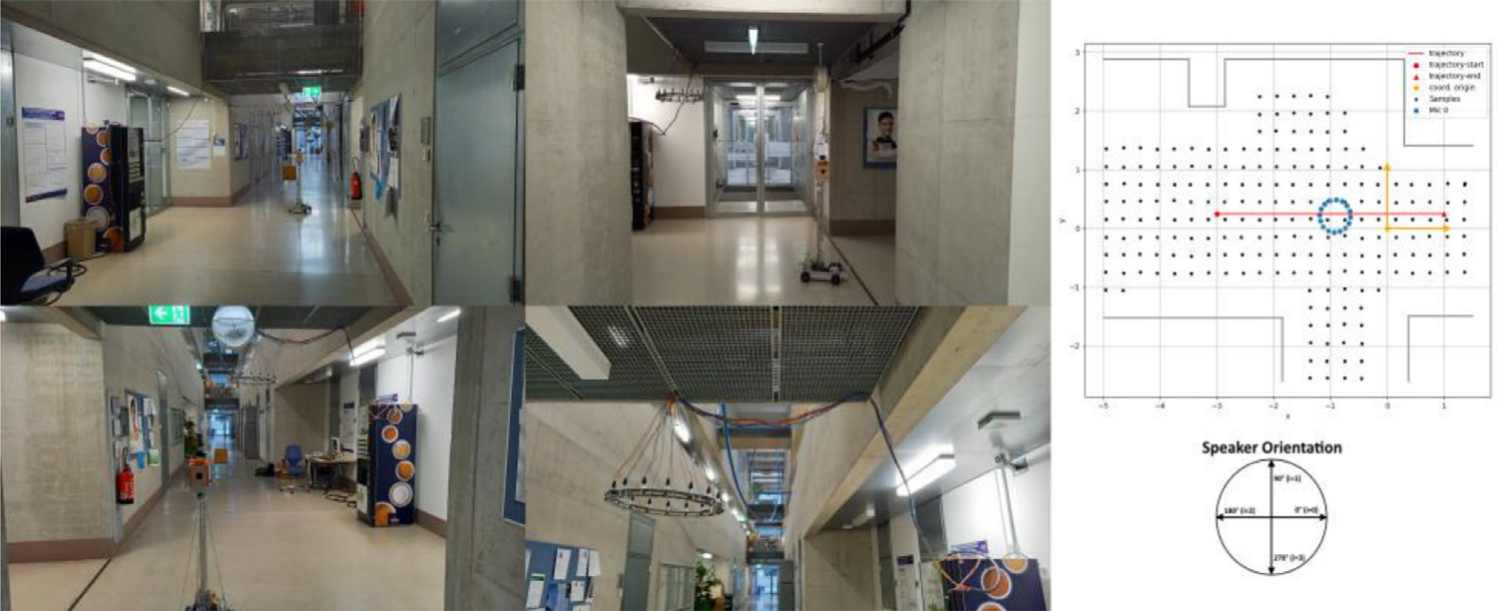
Corridor with autonomous robot (left), Room layout with RIR measurement locations and microphone positions (right).

The corridor was recorded with a grid spacing of 30 cm. The recordings were taken at 210 distinct positions directed towards the four cardinal directions at each position, resulting in a total of 840 RIRs. The corridor is shown in Fig. 2 with the autonomous robot (left) and the geometric layout of the room (right).

The two datasets are stored in the folders data/classroom and data/corridor respectively. Both of them have the same file structure. A dataset directory contains a “meta.json”, an “index” file and multiple “data00x” files (where x is a number starting at 1). The python script **fileio.py** is provided as an interface to easily read the data.

**Fig. 3:**
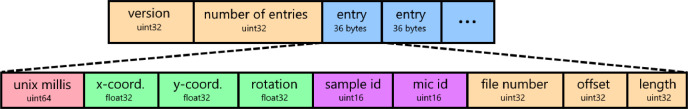
Structure of an index file.
• **Data File:** The data files (“data00x”) contain the RIR data stored as an array with a header (see Figure 4). The recording location and size of the data of a single RIR can be found in the index file. Each RIR is stored as a T×C array, where T is the number of samples (sampled at 48 kHz) and C denotes the number of microphone channels.

• **Meta File:** The file meta.json contains information regarding the microphone arrays and the walls of the room.
• **Index File:** The index file (”index”) contains information about the version of the index file, the number of samples recorded and one entry for each sample recorded (see Fig. 3). Each entry contains a timestamp (**unix millis**, uint64) of when it was recorded. The **x-coord, y-coord** and **rotation** (each float32) contains the position and rotation of the speaker at the time of recording. The direction the speaker was facing is encoded as an integer (**sample id**, uint16). The *x* and *y* coordinates are in millimeters. The rotation is in radiants. The sample id encodes the speaker direction from 0 to 4 for 0° to 270° (e.g.: sample id = 1 ==> rotation = π/2). **Mic_id** (uint16) corresponds to the id of the microphone in the meta file. The location of the recorded sample in the data file is in **file number, offset,** and **length** (each uint32). The file number denotes in which data file to find the sample (”dataXXX” where ”XXX” is the file number, e.g.: “data002” for file number = 2). The offset is the offset in bytes where the stored data for the sample starts and length is the length of the stored sample in bytes.

**Fig. 4:**
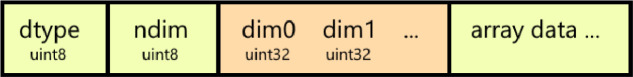
Structure of an array in the data file.

The array format (Fig. 4) starts with a 1 byte (character) representing the datatype of the RIR data (see numpy's dtype.char for more information [3]). The second byte (unsigned integer) represents the number of dimensions of the stored array (always 2). The next *n* bytes represent the size of each dimension, each stored as a 4 byte unsigned integer. The remaining bytes are the data of the array.

## 4 Experimental Design, Materials and Methods

**Robot:**

**Fig. 5:**
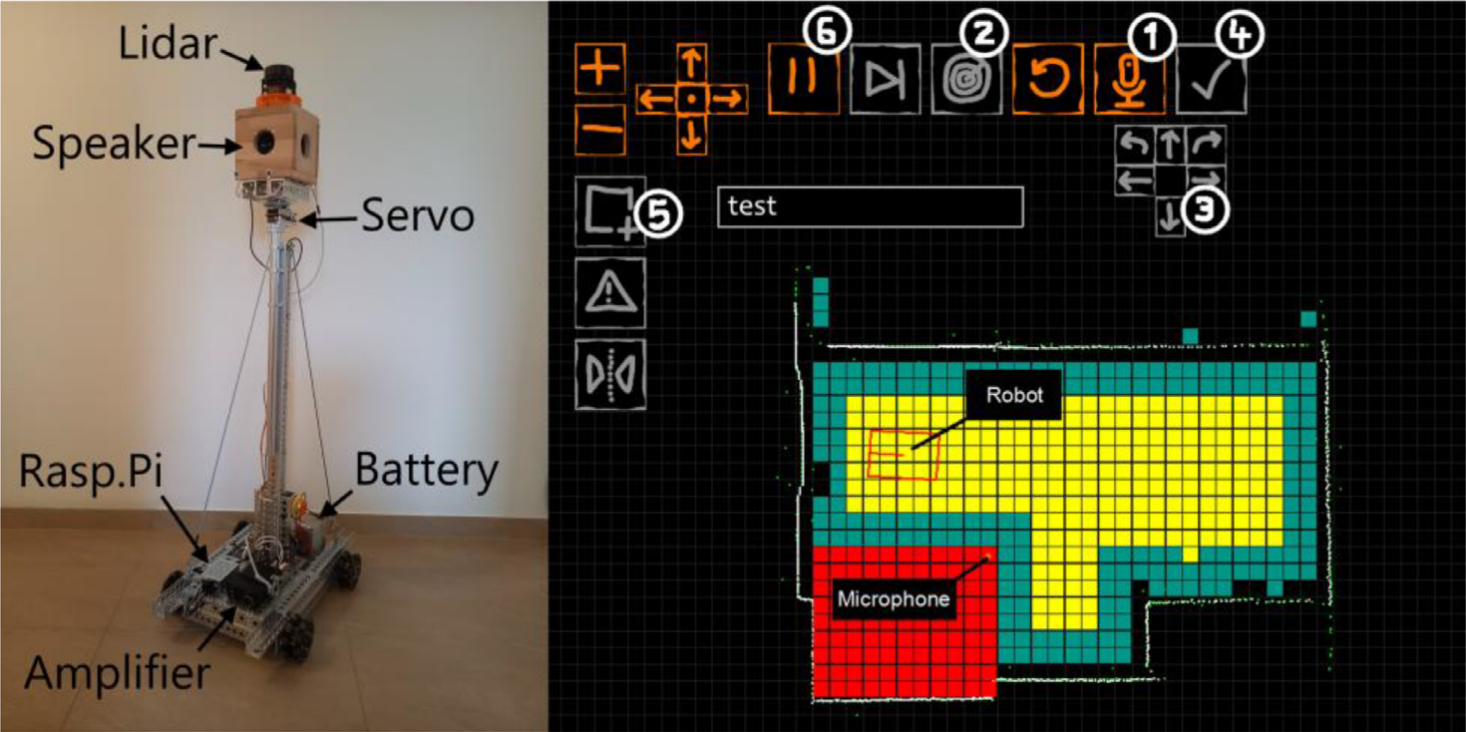
Robot (left), Software GUI with grid (right) (taken from[4]).

The robot (Fig. 5, left) consists of a solid aluminum frame with a pole in the center, which is strapped down by iron wires to ensure mechanical stability. On top of the pole is a servo motor which is used to rotate the platform above. The platform can be rotated from 0° to 360° at a resolution of about 0.1°. A speaker cube is mounted on the platform above the servo, the centers of the speakers are at a height of about 121 cm. The speaker cube has four speakers but only the one in the front is connected and used. On top of the speaker is a 2D-LiDAR sensor (Slamtec RPLIDAR A1) used for navigation. Two geared motors, one on each side at the rear axle, are used to drive the robot. To facilitate a safe turn of the robot, the wheels are swedish 90° wheels. Two of them are attached to each side of each axle. A raspberry pi is used to control the robot. The motors and the amplifier are powered by a 12 V lead acid battery while the raspberry pi, LiDAR and servo are powered by a separate power bank to ensure a measurement campaign for several hours without recharging. In case of insufficient power, the measurement campaign can be continued without any data loss after charging. A more detailed description can be found in [4].

**Software:**

For navigation and the gathering of data the robot is programmed in Python. The Software (Fig. 5, right) provides a visualisation of the recording process as well as the framework to ensure correct localisation and pathfinding of the robot.

Localisation is achieved by ICP-SLAM [4,5]. In particular a reference map of the room based on filtered LiDAR-data is created at the starting position of the robot. This map is partitioned into cells (or a grid) at a resolution provided by the user (see turquoise or yellow cells in Fig. 5, right). In each center of these cells a RIR measurement is performed. Furthermore, the robot autonomously navigates to all cells. The optimal path is found by calculating and comparing the costs of possible paths to the neighboring cells which have not been visited so far. These costs include the estimated turning and driving time. The robot tries to follow the path within certain accuracy bounds.

Upon arrival at a target position, an estimate of the traveled distance and rotation together with a new LiDAR scan is passed to the SLAM algorithm to determine the current position of the robot. The estimate is required to make the SLAM algorithm faster and more accurate. If the driving is not sufficiently accurate, due to the robot mechanics or environmental changes (e.g. floor properties), the robot approaches the destination iteratively, ensuring a precision of about ± 10 mm and ± 0.5° (less accurate in larger spaces). This accuracy can be relaxed as configured in the software.

At every new cell the robot reaches, various audio signals can be played and recorded, here also the angle of the top-mounted platform, hence the speaker direction, can be specified. Several directions of the speaker can be recorded. All the cells which have been visited by the robot are marked in yellow as shown in Fig. 5, right. This process is repeated until every (reachable) cell is visited and RIR measurements have been performed.

In case of unexpected incidents (e.g. robot gets pushed) the robot is not able to match a scan to the previous ones, the user is prompted to align the marked scan to the reference map. This procedure is also used when pausing the recording, e.g. for switching the batteries. The software can be closed during this time, as long as the same room is used after a restart of the system.

As the LiDAR is only able to scan the room at a specific height (about 138 cm), objects like tables etc. might be invisible to the LiDAR. Therefore the user is able to mark areas within the room after the initial scan using the graphical interface (see red areas in Fig. 5, right). The robot will avoid these areas. In addition mirrors and windows can be marked separately, as these would return incorrect distances of the LiDAR system. This procedure can also be used to mark areas where no RIR samples are needed. Marking can happen at any time while the automation is stopped.

**Spatializationof Speech Data:**

For static speakers, spatialization is performed analogous to the image source method [6], i.e.

z(t,m)=s(t)⊛h(t,m),

where h(t,m) denotes the m-th channel of the RIR, and s(t) is the monaural sound source (e.g. clean speech) to convolve it with. Typically, spatialization is done in frequency domain, as the RIRs are relatively long. For static speakers, only a single RIR h(t,m) at the desired speaker location is needed.

**Fig. 6:**
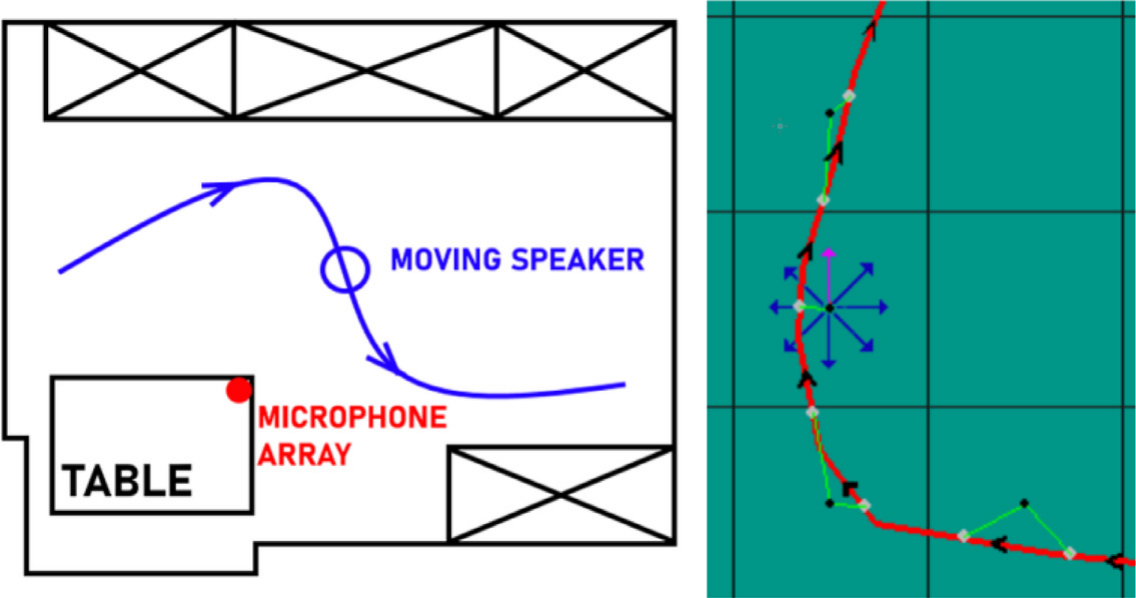
Example trajectory in a room (left), RIR selection for points on trajectory (right).

To simulate moving speakers, we let each speaker follow a virtual trajectory. Fig. 6 shows an example where eight RIRs (distributed evenly among 360°) were measured at the center of each cell. A trajectory is drawn onto the map (red line) which is quantized every 100ms (gray dots). Then, the RIR (black dot at the center of a cell) closest to the quantized point x,y and closest to the direction of the trajectory θ (here the pink arrow) is selected in a nearest-neighbor fashion. This RIR is used for convolution with a monaural speech signal s(t), i.e.

$$u_{x,y,\theta }\left(t,m\right)=s\left(t\right)⊛h_{x,y,\theta }\left(t,m\right),$$

where x and y are the grid indices and θ is the looking direction. Next, we divide the speech signal into B blocks of Δt=100ms length and an overlap of 50ms. For each block b, we select the nearest grid point $x_{b},y_{b}$ and looking direction $\theta_{b}$ of the trajectory at time $t_{b}$. To assemble the final signal z(t,m), we use the overlap-add method [7], i.e.

$$z_{b}\left(m\right)=z_{b}\left(m\right)+u_{x_{b},y_{b},\theta_{b}}\left(t,m\right)w\left(t\right),$$

where $z_{b}\left(m\right)$ denotes a single block of the signal $z(t_{b},...,t_{b}+\Delta t;m)$ at time $t_{b}$, and w(t) is a window function, i.e. Hann [7].

In the repository, we provide a script (spatialization.py) for computing such a spatialization for a given audio file along a specified trajectory.

The python script **spatialization.py** contains functions for spatialization of monoaural audio data. The two files **example1.py** and **example2.py** contain functions for spatializing the two sample audio files **mic_M04_si1038.wav** and **mic_F02_si650.wav** along a trajectory (see Figs. 1 and 2) specified inside the respective functions. The spatialized output of each example are **mic_M04_si1038_spatialized.wav** and **mic_F02_si650_spatialized.wav** respectively**.** The audio samples were taken from [8].

## Limitations

At the moment only two rooms have been recorded. The size of the grid is limited by the runtime of the robot due to battery capacity. The frequency range is limited at the lower end by the size of the loudspeaker that had to be compact for the moving robot.

## Ethics Statement

The authors have read and follow the ethical requirements for publication in Data in Brief.

This work does not involve human subjects, animal experiments, or any data collected from social media platforms.

## CRediT authorship contribution statement

**Stefan Fragner:** Software, Data curation. **Lukas Pfeifenberger:** Software. **Martin Hagmüller:** Data curation, Investigation. **Franz Pernkopf:** Writing – review & editing.

## Data Availability

Dataset of Room Impulse Responses for Realistic Speech Data (Original data) (TU Graz Repository Library & Archives).
